# Direct oral anticoagulant-related medication incidents and pharmacists’ interventions in hospital in-patients: evaluation using reason’s accident causation theory

**DOI:** 10.1007/s11096-021-01302-6

**Published:** 2021-07-02

**Authors:** Hazera Haque, Abdulrhman Alrowily, Zahraa Jalal, Bijal Tailor, Vicky Efue, Asif Sarwar, Vibhu Paudyal

**Affiliations:** 1 School of Pharmacy, College of Medical and Dental Sciences, Sir Robert Aitken Institute for Medical Research, University of Birmingham, Edgbaston, Birmingham, B15 2TT UK; 2 University Hospitals Birmingham NHS Foundations Trust, Birmingham, B15 2TH UK

**Keywords:** Causes, DOAC, Medication incident, Pharmacist intervention, Reason’s accident causation model

## Abstract

*Background* Direct oral anticoagulants (DOACs) have revolutionised anticoagulant pharmacotherapy. However, DOAC-related medication incidents are known to be common. *Objective* To assess medication incidents associated with DOACs using an error theory and to analyse pharmacists’ contributions in minimising medication incidents in hospital in-patients. *Setting* A large University academic hospital in the West Midlands of England. *Methods* Medication incident data from the incident reporting system (48-months period) and pharmacists’ interventions data from the prescribing system (26-month period) relating to hospital in-patients were extracted. Reason’s Accident Causation Model was used to identify potential causality of the incidents. Pharmacists’ intervention data were thematically analysed. *Main outcome measure* (a) Frequency, type and potential causality of DOAC-related incidents; (b) nature of pharmacists’ interventions. *Results* A total of 812 reports were included in the study (124 medication incidents and 688 intervention reports). Missing drug/omission was the most common incident type (26.6%, n = 33) followed by wrong drug (16.1%, n = 20) and wrong dose/strength (11.3%, n = 14). A high majority (89.5%, n = 111) of medication incidents were caused by active failures. Patient discharge without anticoagulation supply and failure to restart DOACs post procedure/scan were commonly recurring themes. Pharmacists’ interventions most frequently related to changes in pharmacological strategy, including drug or dose changes (38.1%, n = 262). Impaired renal function was the most common reason for dose adjustments. *Conclusion* Prescribers’ active failure rather than system errors (i.e. latent failures) contributed to the majority of DOAC-related incidents. Reinforcement of guideline adherence, prescriber education, harnessing pharmacists’ roles and mandating renal function information in prescriptions are likely to improve patient safety.

## Impact of findings on practice statements

- Future interventions to reduce medication errors with DOACs should target active failures such as mistakes and guideline violations.
- Mandating renal function information in prescriptions may help avoid DOACs-related incidents.
- Pharmacist’s clinical checks of DOAC prescriptions are vital as many errors and potential harms are avoided due to their interventions.

## Introduction

Thromboembolic events present major clinical concern. Consequences can be serious, resulting in morbidity or mortality [1]. It is estimated that one in five people die due to causes involving clots [2]. Anticoagulants are first-line therapy for thromboembolic events. They are indicated for prophylaxis and treatment of venous thromboembolism (VTE), including deep vein thrombosis (DVT) and pulmonary embolism (PE). Additionally, they are used to reduce the risk of secondary complications such as stroke in patients with atrial fibrillation (AF) [3, 4]. In recent years, the traditionally used vitamin K antagonist (VKA), warfarin has been gradually replaced by direct oral anticoagulants (DOACs), previously known as novel oral anticoagulants (NOACs).

Currently, there are four DOACs licensed in the United Kingdom (UK) including: dabigatran (direct thrombin inhibitor); and apixaban, rivaroxaban, and edoxaban (factor Xa inhibitors) [3]. The approval of DOACs has revolutionised oral anticoagulation pharmacotherapy and considerably expanded clinical use [5]. DOACs display a preferred safety profile; they have fewer problematic interactions, a fixed-dose regimen and do not require routine international normalised ratio (INR) monitoring, unlike VKAs. Moreover, DOACs have a faster onset effect and a relatively short half-life compared to VKAs. Therefore, anticoagulation effects are achieved quicker [6, 7]. These advantages have encouraged a shift in favour of DOACs in treatment guidelines, consequently increasing national prescribing rates [8].

In this study, a medication incident is defined as a medication related incident or event which actually resulted in or had the potential for a detrimental consequence to a patient [9]. Incidents can occur at any stage of the medication process: prescribing, transcribing, dispensing, administering and monitoring [10]. Previous studies have detected and quantified error types according to the medication process stages [11–13]. Inappropriate prescribing due to incorrect dosing has been highlighted in literature as a major issue in relation to DOAC prescribing [14, 15]. Patient height and weight, baseline activated partial prothrombin time, International Normalized Ratio (INR), full blood count, urea, electrolytes, liver function tests and creatinine clearance (CrCl) are imperative assessments before DOAC initiation [16].

Despite their widespread use, research studying DOAC-related medication incidents is lacking. Though reports of adverse events relating to DOACs and the wider anticoagulant class are available [5, 17–19], analysis of error cause is limited. As they continue to be integrated into clinical practice, a better understanding of the DOAC-related incident types and reasons for occurrence is required. Determining the causes will help identify risk reduction strategies. Theoretical models enable identification of factors contributing to the errors and nature of interventions relevant to addressing the factors. Reason’s Accident Causation Model is a widely used theoretical framework in identifying and understanding medication errors [20].

Errors can be classified into active and latent failures. Active failures are defined as unsafe acts carried out by individuals in direct contact with the patient or system. These can be sub-classified into slips (action-related execution error), lapses (memory-related execution error), mistakes (planning error) and violations (rule-breaking error). Latent failures are system failures that arise from high level organisation decisions [21]. Application of this model and subsequent identification of incident causes will stimulate the basis for future interventions in minimising medication incidents.

Analysing pharmacist interventions during the prescribing process through the use of prescription information databases can enable the understanding of the current roles pharmacists play in mitigating the errors. Databases such as the Prescribing Information and Communications System (PICS), which is an electronic system aimed to provide support for clinical decisions allow such information to be gathered and analysed. Being a communication platform, PICS also allows healthcare professionals to voluntarily log occurrence of events/interventions [22]. The system is designed to minimise medication related errors via various automatic rule-based prescribing checks. Previous studies have successfully used similar information systems to understand nature of errors and communications amongst healthcare professionals around prescribing decisions and mitigating errors [23, 24].

## Aim of the study

The aims of this study were to assess medication incidents associated with DOACs in the hospital in-patients using Reason’s Accident Causation Model and to evaluate the nature of pharmacists’ interventions in minimising DOAC-related medication incidents.

## Ethics approval

This study was approved by the University of Birmingham School of Pharmacy Research Ethics Committee in October 2020 (UoB/SoP/2020–03). The NHS Foundation Trust approved this study as an audit (CARMS-16618) and no further NHS ethical approval was required.

## Method

A two-phased study was conducted. Firstly, medication incidents reported on DATIX, over a 48-month period (September 2016–September 2020), by healthcare professionals were analysed. DATIX is a widely used, web-based, voluntary incident reporting and risk management system. This database collates occurrence of all events that have resulted in or have the potential to result in patient safety incidents [25]. Secondly, pharmacists’ interventions submitted to the PICS over a 26-month period (August 2018 to September 2020), were reviewed.

## Setting

Both databases, DATIX and PICS were obtained from one of the largest teaching hospitals in England with 1383 beds. It receives approximately 65,000 in-patient admissions in a given year [26]. The hospital utilises electronic prescribing systems for all prescribing activities [27]. Clinical pharmacy service is available in all wards where clinical pharmacists and pharmacy technicians provide ward cover on a daily basis including medicines reviews and reconciliation. Patients when admitted undergo drug history with a clinical pharmacist or a technician after which a pharmacist undertakes medicines reconciliation and medicines optimisation in collaboration with other healthcare team until the point of discharge.

## Data processing and analysis

Search terms (DOAC, NOAC, apixaban, rivaroxaban, dabigatran, edoxaban and anticoagulant) were used to extract relevant data from both databases. The acquired data were processed on Microsoft Excel in an anonymous form. Then, data were filtered according to the inclusion criteria: adult patients (≥ 18 years old) who were prescribed a DOAC. Data cleaning was used to remove duplicate records, incomplete and unclear information. Quantitative analysis was used to investigate the identified medication incidents from DATIX. Categorisation according to incident type was conducted primarily by one author (HH) followed by independent checks by two authors (VP and ZJ). Classification of categories was determined by identifying the common reoccurring events. Descriptive statistics including frequency and percentages were used to analyse the data.

**Fig. 1:**
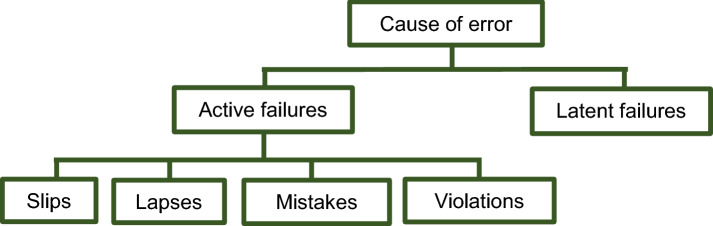
Accident Causation Model classification system

Reason’s Accident Causation Model (Fig. 1) was used to determine the contributory factors associated with medication incidents and to ultimately establish potential causality. The free text data from the DATIX database were examined to classify causes of medication incidents according to the model categories. Organisation into sub-categories dependent on the most common themes was conducted to enable further investigation. Quantitative analysis via descriptive statistics was performed to determine the major cause of medication incidents.

Data from the PICS database in relation to pharmacist interventions and associated rationale were classified according to the nature of the intervention. The classification system used was adapted from a previous study [28]. These included pharmacological strategy such as drug change or patient education. Two additional categories: ‘documentation’ and ‘other’ were also added. Sub-categories were included as appropriate.

## Results

### Evaluation of DOAC incidents

A total of 419 incidents were identified over a 48-month period from the initial DATIX system search. However, 241 incidents were excluded due to the reports not being DOAC-related (i.e., regarding warfarin, enoxaparin, tinzaparin), duplicate records and incomplete information (i.e., DOAC unspecified, use of unclear abbreviations). Of the remaining 178 DOAC-related incidents, a further 54 cases were excluded as they were not deemed as medication incidents. For instance, these were concerning access, transfer and cancellation of procedures. Hence, 124 reports were included in this study following inclusion and exclusion filtering.

**Fig. 2:**
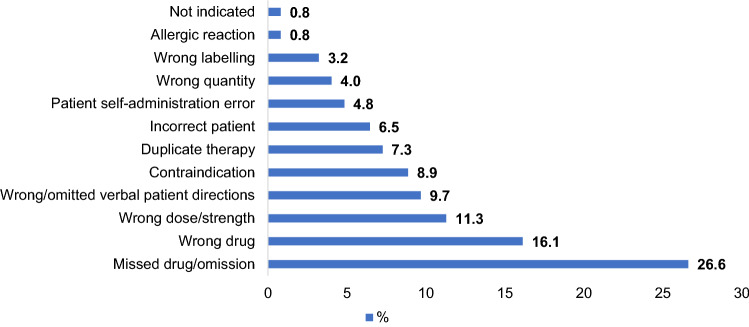
Medication incidents categorised according to incident type (%)

A number of factors resulted in medication incidents as shown in Fig. 2. The majority of the incidents occurred during the prescribing and administration stage of the medication process. The most common errors resulting in an incident were missing drug/omission (drug not prescribed, administered or missed dose) (26.6%, n = 33), wrong drug (16.1%, n = 20) and wrong dose/strength (11.3%, n = 14). Table 1 shows the contributory factors that resulted in medication incidents in line with Reason’s Accident Causation Model. Almost all (89.5%, n = 111) medication incidents were classified as active failures. The active failures comprised of lapses (29.8%, n = 37), slips (24.2%, n = 30), mistakes (22.6%, n = 28) and violations (12.9%, n = 16). The rest of the incidents were classified as latent failures (10.5%, n = 13). These categories were sub-categorised, as summarised in Table 1.

**Table 1:** Contributory factors to medication incidents based on Reason’s Accident Causation Model

Error cause, n = 124	Error	% (n)*
Active failures (slips), n = 30	*Dispensing error*	
	Look-alike sound-alike medications	6.7 (2)
	Selecting wrong drug	16.7 (5)
	Selecting wrong dose	13.3 (4)
	Wrong labeling	10 (3)
	Wrong quantity	6.6 (2)
	Incorrect patient	26.7 (8)
	Others	20 (6)
Active failures (lapses), n = 37	*Lack of plan adherence*	
	Omission	48.6 (18)
	Failure to restart drug	10.8 (4)
	Failure to discontinue drug	10.8 (4)
	Omitted verbal patient directions	16.2 (6)
	Others	13.5 (5)
Active failures (mistakes), n = 28	*Drug prescribing error*	
	Contraindication	14.3 (4)
	Unlicensed indication	3.6 (1)
	No clear indication	7.1 (2)
	Allergic reaction	3.6 (1)
	Duplicate therapy	14.3 (4)
	*Dose prescribing error*	
	Contraindication	10.7 (3)
	Wrong dose on admission	3.6 (1)
	Wrong dose for indication	28.6 (8)
	Drug administration despite procedure booking	7.1 (2)
	Others	7.1 (2)
Active failures (violations), n = 16	*Non-compliance to policy*	
	Prescribing without confirmed diagnosis	6.3 (1)
	Not using the most up to date TTO	18.8 (3)
	Not sending RICaD** to anticoagulation team	12.5 (2)
	Others	12.5 (2)
	*Patient related*	
	Medication stoppage	12.5 (2)
	Unauthorised self-medication	31.3 (5)
	Not taking as instructed	6.3 (1)
Latent failures, n = 13	*Inadequate training/knowledge*	
	Failure to administer as unaware of stock storage	15.4 (2)
	Wrong patient directions	46.2 (6)
	Duplicate dose to overcome missed dose	7.7 (1)
	*Insufficient communication/handover*	
	Duplicate dose administration	15.4 (2)
	Missed dose	7.7 (1)
	Duplicate therapy	7.7 (1)

*Rounding to one decimal place, therefore may not exactly add to 100%

** Rationales for Initiation, Continuation and Discontinuation (RICaD) form; TTO: to take out (prescriptions)

#### Missing drug/omission

Various scenarios resulted in drug dose omission, each with differing error causes as defined by Reason’s Accident Causation Model (see Table 1). The majority of drug omission incidents were due to lapses including lack of plan adherence (48.6%, n = 18). A reoccurring theme was patient discharge from hospital without anticoagulation supply. Failure to restart DOAC post procedure/scan was also a common cause resulting in drug omission (10.8%, n = 4). A few cases of drug omission due to violation concerned to take out (TTO) prescriptions which had inadvertently not been updated by the prescriber prior to patient discharge (18.8%, n = 3). Latent failures resulting in drug dose omission involved insufficient team communication/handover (7.7%, n = 1).

#### Wrong drug

Medication incidents due to wrong drug supply comprised a high percentage of incidents. Causes of error were largely due to slips and mistakes (Table 1). Slips involved dispensing errors such as selecting the wrong drug due to incorrect system/clerking documentation (16.7%, n = 5). There were two reported cases where the look-alike, sound-alike drug rosuvastatin was dispensed instead of rivaroxaban (6.7%, n = 2). A large proportion of slips involved drug supply to incorrect patients (26.7%, n = 8).

#### Wrong dose/strength

This category included wrong strength of the drug being prescribed (for the indication), wrong strength being dispensed or duplicate dose administration (due to insufficient handover or duplicate dose to overcome missed dose). The most common dose/strength related medication incident was the prescribing of wrong dose for the given indication (28.6%, n = 8). This error is classified as a mistake (see Table 1). For example, a patient diagnosed with left leg DVT was commenced on rivaroxaban 15 mg once daily. However, the patient should have been prescribed 15 mg twice daily for the first 21 days as per national guidance [3]. Latent failures resulting in wrong dose/strength supply involved the double dose administration of DOAC to overcome the effect of missed doses (15.4%, n = 2).

## Evaluation of pharmacist interventions

Following the initial PICS database search, a total of 1024 pharmacist interventions were identified over a 26-month period. A total of 336 intervention cases were excluded from the study as they were not DOAC-related, or information was unclear and incomplete. The remaining 688 submitted interventions specific to DOACs formed the data sample included in this study.

Changes in pharmacological strategy comprised the highest proportion (38.1%, n = 262). It was followed by interventions related to quantity of drug (26.5%, n = 182) and those related to patient education (14.5%, n = 100) (see Table 2). Start/restart of DOACs accounted for more than half of the pharmacological strategy interventions (51.5%, n = 135) (Table 2). Drug change was the second most common pharmacological strategy intervention (21.0%, n = 55). Almost all of the quantity of drug interventions were associated with DOAC dose changes (91.2%, n = 166). The rationale for the interventions varied, as shown in Table 2.

**Table 2:** Rationale for pharmacist interventions

Intervention category	Subcategory	Reasons for intervention	n (%)	Examples of interventions made by pharmacists
Quantity of drug, 26.5%, n = 182	Dose change	Age	38 (16.7)	Rivaroxaban dose reduced to 15 mg OD as recorded GFR 29 (CrCl 49) Edoxaban dose increased to 60 mg OD as eGFR improved (GFR 62, CrCl > 50 ml/min) Changed from dabigatran 150 mg to 110 mg to match medicines reconciliation
		Renal function	67 (29.4)	
		Weight	41 (18.0)	
		Adverse effect (bleeding)	2 (0.9)	
		Pre-admission dose	9 (3.9)	
		Indication/per guidelines	30 (13.2)	
		To match medicine reconciliation	10 (4.4)	
		Not specified	31 (13.6)	
		Total	228*	
	Change schedule	Total	1	Apixaban timings altered so patient wouldn’t miss a day of treatment
	Change duration of treatment	Per guidelines	7 (46.7)	Apixaban duration of loading dose corrected to 7 days
		Not specified	8 (53.3)	
		Total	15	
Pharmacological strategy, 38.1%, n = 262	Drug change	Interaction	7 (12.7)	Interaction between voriconazole and apixaban. Patient to be switched to warfarin Advised doctor that apixaban less effective if weight > 120 kg, warfarin more suitable Apixaban switched to enoxaparin due to swallowing issues Tinzaparin switched to edoxaban to improve compliance post-discharge
		Surgery	3 (5.5)	
		More effective option available	2 (3.6)	
		Renal function	7 (12.7)	
		Per history (Hx)	6 (10.9)	
		Dysphagia	2 (3.6)	
		Aid compliance	1 (1.8)	
		Not specified	27 (49.1)	
		Total	55	
	Change administration	Dysphagia	4 (66.7)	Rivaroxaban paused due to dysphagia. Advised that the patient could continue, crush and disperse in water
		Other	2 (33.3)	
		Total	6	
	Start/restart medication	Pre-admission	25 (18.5)	Proposed pre-admission dabigatran Proposed rivaroxaban to start 72 h post-surgery as per procedure Newly diagnosed AF. Team to consider starting apixaban + anticoagulation referral
		Discharge	30 (22.2)	
		Post-procedure	9 (6.7)	
		Diagnosis	10 (7.4)	
		Post-scan	4 (3.0)	
		Not specified	57 (42.2)	
		Total	135	
	Medication paused	Surgery	9 (31.0)	Advised to stop edoxaban for 24-48 h prior to surgery Apixaban was being withheld as patient has a subdural haematoma Patient at high risk of falling—apixaban paused
		Reduced renal function	5 (17.2)	
		Active bleeding	3 (10.3)	
		Fall risk	2 (6.9)	
		Vomiting	1 (3.4)	
		Not specified	9 (31.0)	
		Total	29	
	Discontinue medication	Interaction	1 (3.4)	Asked doctor to remove Ibuprofen from TTO due to high risk of bleeding with Apixaban Apixaban stopped due to small subarachnoid haemorrhage Rivaroxaban stopped due to risk of falls
		Duplicate therapy	3 (10.3)	
		Active bleeding	4 (13.8)	
		Bleeding risk	2 (6.9)	
		Fall risk	1 (3.4)	
		Renal impairment	3 (10.3)	
		Not indicated	3 (10.3)	
		Not specified	12 (41.4)	
		Total	29	
	Monitoring	Interaction	3 (37.5)	Apixaban needs reviewing, patient’s LFTs not within range – Doctor to monitor Advised GP to review renal function in one week’s time to check GFR
		Liver impairment	1 (12.5)	
		Monitor renal function	1 (12.5)	
		Intolerance	1 (12.5)	
		Other	2 (25.0)	
		Total	8	
Patient education, 14.5%, n = 100	Enhance compliance	Change dosing regime	1 (50.0)	Advised to see if switching from apixaban 10 mg OD to rivaroxaban is an option to help with compliance without imposing a risk
		Patient refusal to take medicine	1 (50.0)	
		Total	2	
	Newly initiated	Diagnosis	3 (15.8)	Conversation with patient about change from apixaban to edoxaban, patient thought 60 mg (edoxaban) was too high a dose in comparison to 5 mg (apixaban)
		Drug change	2 (10.5)	
		Not specified	14 (73.7)	
		Total	19	
	General counselling	Total	79	
Documentation, 9.7%, n = 67	Update drug record	Document end date	1 (6.3)	Informed doctor of patient’s regular medications to be charted, including apixaban
		Amend drug	2 (12.5)	
		Amend dose	4 (25.0)	
		Drug missing from chart	5 (31.3)	
		Other	4 (25.0)	
		Total	16	
	Update discharge letter	Drug change	4 (30.8)	Discharge letter needed to be updated to include that warfarin has been switched to apixaban
		Amend dose	3 (23.1)	
		Other	6 (46.2)	
		Total	13	
	Indication	Total	23	Confirmed indication for apixaban as not clearly documented in the discharge letter
	Thrombosis assessment update	Total	15	Thrombosis assessment—contraindication to enoxaparin as patient on rivaroxaban
Other, 11.2%, n = 77	Book follow-up appointment	Total	5	Advised doctor that the patient was new to apixaban and will need anticoagulation appointment referral on discharge
	Check dose	Doesn’t comply with guidelines	4 (18.2)	Queried why lower dose of apixaban prescribed as patient did not meet criteria for dose reduction in AF
		Subtherapeutic	7 (31.8)	
		Renal function	4 (18.2)	
		Other	7 (31.8)	
		Total	22	
	Consult prescriber	Rational for drug change	2 (4.0)	Queried with the doctor about restarting rivaroxaban as bleeding has settled Discussed with doctor about restarting apixaban—renal function shows slight improvement
		When to restart drug	14 (28.0)	
		Rational for drug choice	4 (8.0)	
		Rational for drug discontinuation	3 (6.0)	
		Rational for duplicate therapy	12 (24.0)	
		Query drug duration	1 (2.0)	
		Review plan	14 (28.0)	
		Total	50	

*The total number of reasons for dose change does not equal the number of dose change intervention cases (n = 166) due to multifactorial rationale (i.e., dose change for one patient due to both age and weight); *AF* atrial fibrillation, *GFR* glomerular filtration rate, *GP* general practitioner, *LFTs* liver function test, *OD* once daily’ *TTO* to take out (prescriptions)

## Dose adjustments

Interventions owing to inappropriate dose prescribing contributed to the largest overall percentage of recorded interventions in relation to dose adjustments (Table 2). In many circumstances, multifactorial rationale including age, weight and renal function were assessed to establish suitable doses. Renal function was the most common reason for dose adjustment (29.4%, n = 67). The majority of these cases involved renally impaired patients requiring dose reductions and a few related to dose increase as renal function improved. Age and weight considerations also led to the dose adjustments (16.7%, n = 38 and 18.0%, n = 41 respectively). Dose modification interventions (13.2%, n = 30) were related to indication and/or treatment guidelines such as the switch from initiation to maintenance doses or changing between prophylactic and therapeutic doses.

## Start/restart medication

Key rationale for this intervention included the initiation or re-initiation of DOAC therapy on discharge (22.2%, n = 30). A common scenario involved in-patient low molecular weight heparin therapy and re-initiation of DOACs on discharge, in line with the hospital Trust policy guidelines [29]. New diagnosis of thromboembolic indications, such as AF and PE resulted in the initiation of appropriate DOAC therapy (7.4%, n = 10). Restarting anticoagulation post-procedure or post-scan comprised of 6.7% (n = 9) and 3.0% (n = 4) respectively (see Table 2).

## Drug change

Foundation for changes in anticoagulation therapy involved drug-drug interactions (12.7%, n = 7). Concurrent use with antibiotics (i.e., rifampicin) or antifungals (i.e., voriconazole) comprised almost all of the recorded DOAC interactions. A total of 12.7% (n = 7) of drug change interventions involved contraindication due to renal impairment. Further significant rationale included dysphagia (3.6%, n = 2), contraindication due to surgery (5.5%, n = 3) and more effective treatment (3.6%, n = 2), as summarised in Table 2.

## Patient education

General counselling formed the majority of patient education interventions (79%, n = 79). Also, 19% (n = 19) of interventions were related to patients newly initiated on a DOAC. The remaining 2% (n = 2) concentrated on enhancing patient compliance (see Table 2).

## Discussion

### Key findings

This study shows that the majority of the DOAC-related incidents in the hospital in-patients occurred in the prescribing and administration stages of the medication process. Missing drug/omission was the most common incident type and the majority of medication incidents were caused by active failures. Patient discharge without anticoagulation supply and failure to restart DOACs post procedure/scan were commonly recurring themes. Pharmacist interventions most frequently related to changes in pharmacological strategy, including drug or dose changes, often in response to impaired renal function.

The findings of this study are in line with previous studies which reported a high degree of anticoagulant incidents due to inappropriate prescribing and administration, [18, 30] and high rates of drug omissions [31]. New insights from the application of Reason's Accident Causation Model in this study, however, suggested that most of the errors were due to active failures (lapses, slips, mistakes and violations). As causes of the medication incidents were largely due to the performance of the healthcare professionals, rather than faults in system or the organisation, there is a clear need to support healthcare professionals in guideline adherence and minimising active failures. Access to succinct, user-friendly prescribing guidelines and decision support tools are imperative to aid prescribing.

A detailed analysis of dose change rationale advised by pharmacists related to renal function as the top cause. The National Patient Safety Agency (NPSA) in the UK has warranted a safety alert with regard to inappropriate anticoagulant dose prescribing, particularly concerning renal function [4]. Dose adjustments according to renal function is highly important to ensure optimal thromboembolic therapy whilst reducing the associated bleeding risks. The Medicines and Healthcare products Regulatory Agency (MHRA) advises calculation of creatinine clearance prior to making dosing decisions [32]. These factors highlight that incidence of inappropriate renal dosing is a common occurrence and suggest the need for measures such as mandating renal function information in prescriptions [11] to reduce related incidents.

Previous research demonstrates that multifaceted interventions combining educational and technological support to healthcare professionals are effective in reducing prescribing errors. Outreach based educational interventions to other healthcare professionals and assisted by technology that emphasise guideline adherence have been shown to be effective in minimising errors [33]. Continuous professional development training and assessment opportunities for healthcare professionals in relation to correct prescribing and administration of DOACs are needed. Workload, stress, time pressured consultations and busy working environment have been shown to contribute to active failures such as slips and lapse and as such organisational support and effective team working can address such barriers. While electronic prescribing systems are likely to minimise errors, it has been reported that certain errors are likely features of electronic prescribing systems [34].

Our findings show that pharmacists play an integral role in minimising medication incidents. Some key interventions included dose and drug alterations, stopping and starting treatment, documentation and patient counselling. Overall, dose changes contributed to the highest percentage of recorded interventions. This is consistent with several published studies investigating pharmacist interventions in other therapeutic areas [35–38]. A recent meta-analysis of pharmacists’ interventions such as prescription review, educational sessions delivered to other healthcare professionals and attendance in clinical rounds could reduce medication errors by as much as three quarters [39].

## Strengths and limitations

Large, comprehensive data samples were extracted over a substantial timeframe using sophisticated incident and intervention reporting databases. The commonly applied framework, Reason’s Accident Causation Model was used providing indication of error causality allowing identification of areas of improvement for patient safety. However, categorisation using this model can be subjective particularly when there is a lack of adequate free text information available. In particular, system-related factors are likely to be underreported due to known barriers of reporting medication errors including fear and accountability [40]. In addition, both reporting systems operate voluntary. Underreporting, selective and incomplete reporting are recognised; our results are likely to be underestimated compared to the true values. Also, data were obtained from only one large hospital Trust in the UK limiting generalisability. This study used a theoretical model to allow analysis and interpretation of the data in a structured way, which may enable other researchers to classify DOAC-related incidents and interventions accordingly.

## Recommendations for research

Future observational research can be conducted to overcome bias in the voluntary reporting system. Qualitative studies consisting of semi-structured interviews of patients, nurses, prescribers and pharmacists to further explore causes and ways to mitigate DOACs medication incidents are needed. Additional research should aim to extend the scope of this study to incident severity and its impact on patient health outcomes. Development and evaluations of interventions to minimise errors are needed. Research should be extended to non-hospital settings.

## Conclusion

Prescribers’ active failure rather than system errors (i.e. latent failures) contributed to the majority of DOAC-related incidents in hospital settings. It is important to stress the importance of guideline adherence to healthcare professionals, in particular ensuring renal function assessment to determine appropriate dosing schedules. Mandating renal function information in prescriptions is recommended to allow ease of checking. As pharmacists play a crucial role in minimising incidents at present, additional strategies such as strengthening clinical governance, pharmacists’ involvement in the on-going training of staff and annual staff assessments are required to improve patient safety in relation to DOACs.

## References

[1] Mekaj YH, Mekaj AY, Duci SB, Miftari EI (2015). New oral anticoagulants: their advantages and disadvantages compared with vitamin K antagonists in the prevention and treatment of patients with thromboembolic events. Therap Clin Risk Manag.

[2] Thrombosis UK. Thrombosis Statistics. 2020; https://thrombosisuk.org/thrombosis-statistics.php. Accessed 10 Nov 2020.

[3] Joint Formulary Committee (2020). British National Formulary.

[4] National Institute for Health and Clinical Excellence. Anticoagulants, including direct-acting oral anticoagulants (DOACs). [KTT16]. *Nice Guidance: key therapeutic topic*. 2019; Available at: https://www.nice.org.uk/advice/ktt16/chapter/Evidence-context. Accessed 10 Nov 2020.

[5] Barr D, Epps QJ (2019). Direct oral anticoagulants: a review of common medication errors. J Thromb Thrombolysis.

[6] Vinogradova Y, Coupland C, Hill T, Hippisley-Cox J (2018). Risks and benefits of direct oral anticoagulants versus warfarin in a real world setting: cohort study in primary care. BMJ.

[7] Chen A, Stecker E, Warden AB (2020). Direct oral anticoagulant use: a practical guide to common clinical challenges. J Am Heart Assoc.

[8] Ho KH, van Hove M, Leng G (2020). Trends in anticoagulant prescribing: a review of local policies in English primary care. BMC Health Serv Res.

[9] Cumbria, Northumberland, Tyne and Wear NHS Foundation Trust. Appendix 2, Incident Policy Practice Guidance Note Medication Incidents. 5(2). IP-PGN 07 (CNTW(O)05). 2019; Available at: https://www.cntw.nhs.uk/content/uploads/2016/04/IP-PGN-07-Medication-Incidents-V05-Iss-2-Oct19-1.pdf. Accessed 25 Nov 2020.

[10] Vogenberg FR, Benjamin D (2011). The medication-use process and the importance of mastering fundamentals. Pharm Ther.

[11] Viprey M, Jeannin R, Piriou V, Chevalier P, Michel C, Aulagner G (2017). Prevalence of drug-related problems associated with direct oral anticoagulants in hospitalized patients: a multicenter, cross-sectional study. J Clin Pharm Ther.

[12] Howard M, Lipshutz A, Roess B, Hawes E, Deyo Z, Burkhart JI (2017). Identification of risk factors for inappropriate and suboptimal initiation of direct oral anticoagulants. J Thromb Thrombolysis.

[13] Tran E, Duckett A, Fisher S, Bohm N (2017). Appropriateness of direct oral anticoagulant dosing for venous thromboembolism treatment. J Thromb Thrombolysis.

[14] Whitworth MM, Haase KK, Fike DS, Bharadwaj RM, Young RB, MacLaughlin EJ (2017). Utilization and prescribing patterns of direct oral anticoagulants. Int J Gen Med.

[15] Larock A, Mullier F, Sennesael A, Douxfils J, Devalet B, Chatelain C (2014). Appropriateness of prescribing dabigatran etexilate and rivaroxaban in patients with nonvalvular atrial fibrillation: a prospective study. Ann Pharmacother.

[16] Wirral University Teaching Hospital NHS Foundation Trust. Oral Anticoagulants (VKA and DOAC) Guidelines for prescribing, monitoring and management. 2016; Available at: https://www.sps.nhs.uk/wp-content/uploads/2018/02/oral-anticoagulant-oral-guidelines-for-prescribing-monitoring-and-management-v41-2s1.4d-and-2s1.5v-and-5s1.1.d.pdf. Accessed 25 Nov 2020.

[17] National Pharmacy Association. Medicines Safety Officer (MSO) update Quarter 2 2019. Available https://www.npa.co.uk/news-and-events/news-item/medicines-safety-officer-mso-update-quarter-2-2019/. Accessed 27 May 2021.

[18] Dreijer AR, Diepstraten J, Bukkems VE, Mol PG, Leebeek FW, Kruip MJ (2019). Anticoagulant medication errors in hospitals and primary care: a cross-sectional study. Int J Qual Health Care.

[19] Fanikos J, Stapinski C, Koo S, Kucher N, Tsilimingras K, Goldhaber SZ (2004). Medication errors associated with anticoagulant therapy in the hospital. Am J Cardiol.

[20] Elliott M, Page K, Worrall-Carter L (2012). Reason’s accident causation model: application to adverse events in acute care. Contemp Nurse.

[21] Moyen E, Camiré E, Stelfox HT (2008). Clinical review: medication errors in critical care. Crit Care.

[22] University Hospital Birmingham NHS Foundation Trust. Birmingham Systems PICS. 2012; Available at: https://www.uhb.nhs.uk/birmingham-systems-pics.htm. Accessed 28 Nov 2020.

[23] Redwood S, Rajakumar A, Hodson J, Coleman JJ (2011). Does the implementation of an electronic prescribing system create unintended medication errors? A study of the sociotechnical context through the analysis of reported medication incidents. BMC Med Inform Decis Mak.

[24] De Waal S, Lucas L, Ball S, Pankhurst T. Dietitians can improve accuracy of prescribing by interacting with electronic prescribing systems. BMJ Health Care Inform. 2019;26:e000019. 10.1136/bmjhci-2019-000019. 10.1136/bmjhci-2019-000019 PMC7062321 31201200

[25] Black Country Partnership NHS Foundation Trust. Reporting an incident SOP1. 2019; Available at: https://www.bcpft.nhs.uk/about-us/our-policies-and-procedures/i/858-incident-reporting-sop-1-reporting-an-incident/file?tmpl=component. Accessed 28 Nov 2020.

[26] University Hospitals Birmingham. Freedom of information requests. FOI 0093HGS/0053UHB Beds. Available: https://hgs.uhb.nhs.uk/foi-0093hgs-0053uhb-beds/. Accessed 29 June 2021.

[27] University Hospitals Birmingham NHS Foundation Trust. About us. 2020; Available at: https://www.uhb.nhs.uk/about-us.htm. Accessed 10 Dec 2020.

[28] Faus MJ, Sabater-Hernández D, Amariles P (2007). Types of pharmacist interventions intended to prevent and solve negative outcomes associated with medication. Pharmacotherapy.

[29] University Hospital Birmingham NHS Foundation Trust. Prevention and Treatment of Venous Thromboembolism (VTE) Policy. 2019; https://www.uhb.nhs.uk/Downloads/pdf/controlled-documents/VtePreventionPolicy.pdf. Accessed 2 Dec 2020.

[30] Jovanovska T, Fitzsimons K, Ferguson C, Koay A (2019). Types and causes of anticoagulant-related medication incidents across hospitals in Western Australia. J Pharm Pract Res.

[31] Cousins DH, Gerrett D, Warner B (2012). A review of medication incidents reported to the national reporting and learning system in England and Wales over 6 years (2005–2010). Br J Clin Pharmacol.

[32] GOV.UK. Prescribing medicines in renal impairment: using the appropriate estimate of renal function to avoid the risk of adverse drug reactions. 2019; https://www.gov.uk/drug-safety-update/prescribing-medicines-in-renal-impairment-using-the-appropriate-estimate-of-renal-function-to-avoid-the-risk-of-adverse-drug-reactions. Accessed 8 Dec 2020.

[33] Avery AJ, Rodgers S, Cantrill JA, Armstrong S, Cresswell K, Eden M (2012). A pharmacist-led information technology intervention for medication errors (PINCER): a multicentre, cluster randomised, controlled trial and cost-effectiveness analysis. Lancet.

[34] Lewis PJ, Ashcroft DM, Dornan T, Taylor D, Wass V, Tully MP (2014). Exploring the causes of junior doctors' prescribing mistakes: a qualitative study. Br J Clin Pharmacol.

[35] Falcão F, Viegas E, Lopes C, Branco R, Parrinha A, Alves ML (2015). Hospital pharmacist interventions in a central hospital. Eur J Hosp Pharm.

[36] Halvorsen KH, Ruths S, Granas AG, Viktil KK (2010). Multidisciplinary intervention to identify and resolve drug-related problems in Norwegian nursing homes. Scand J Prim Health Care.

[37] Reis WCT, Scopel CT, Correr CJ, Andrzejevski VMS (2013). Analysis of clinical pharmacist interventions in a tertiary teaching hospital in Brazil. Einstein (Sao Paulo).

[38] Somers A, Robays H, De Paepe P, Van Maele G, Perehudoff K, Petrovic M (2013). Evaluation of clinical pharmacist recommendations in the geriatric ward of a Belgian university hospital. Clin Interv Ageing.

[39] Naseralallah N, Hussain LM, Jaam TA, Pawluk MSA (2020). Impact of pharmacist interventions on medication errors in hospitalized pediatric patients: a systematic review and meta-analysis. Int J Clin Pharm.

[40] Vrbnjak D, Denieffe S, O’Gorman C, Pajnkihar M (2016). Barriers to reporting medication errors and near misses among nurses: A systematic review. Int J Nurs Studies.

